# Designing and In Vitro Characterization of pH-Sensitive Aspartic Acid-Graft-Poly(Acrylic Acid) Hydrogels as Controlled Drug Carriers

**DOI:** 10.3390/gels8080521

**Published:** 2022-08-19

**Authors:** Muhammad Suhail, Chih-Wun Fang, I-Hui Chiu, Ming-Chia Hung, Quoc Lam Vu, I-Ling Lin, Pao-Chu Wu

**Affiliations:** 1 School of Pharmacy, Kaohsiung Medical University, 100 Shih-Chuan 1st Road, Kaohsiung 80708, Taiwan; 2 Divison of Pharmacy, Zuoying Branch of Kaohsiung Armed Forces General Hospital, Kaohsiung 81342, Taiwan; 3 Department of Clinical Pharmacy, Thai Nguyen University of Medicine and Pharmacy, 284 Luong Ngoc Quyen Str., Thai Nguyen 24000, Vietnam; 4 Department of Medicine Laboratory Science and Biotechnology, College of Health Science, Kaohsiung Medical University, Kaohsiung 80708, Taiwan; 5 Department of Medical Research, Kaohsiung Medical University Hospital, Kaohsiung 80708, Taiwan; 6 Drug Development and Value Creation Research Center, Kaohsiung Medical University, Kaohsiung 80708, Taiwan

**Keywords:** aspartic acid, hydrogels, swelling study, and acetaminophen

## Abstract

Acetaminophen is an odorless and white crystalline powder drug, used in the management of fever, pain, and headache. The half-life of acetaminophen is very short; thus, multiple intakes of acetaminophen are needed in a day to maintain a constant pharmacological action for an extended period of time. Certain severe adverse effects are produced due to the frequent intake of acetaminophen, especially hepatotoxicity and skin rashes. Therefore, a drug carrier system is needed which not only prolongs the release of acetaminophen, but also enhances the patient compliance. Therefore, the authors prepared novel aspartic acid-graft-poly(acrylic acid) hydrogels for the controlled release of acetaminophen. The novelty of the prepared hydrogels is based on the incorporation of pH-sensitive monomer acrylic acid with polymer aspartic acid in the presence of ethylene glycol dimethacrylate. Due to the pH-sensitive nature, the release of acetaminophen was prolonged for an extended period of time by the developed hydrogels. Hence, a series of studies was carried out for the formulated hydrogels including sol-gel fraction, FTIR, dynamic swelling, polymer volume analysis, thermal analysis, percent porosity, SEM, in vitro drug release studies, and PXRD analysis. FTIR analysis confirmed the grafting of acrylic acid onto the backbone of aspartic acid and revealed the development of hydrogels. The thermal studies revealed the high thermal stability of the fabricated hydrogels as compared to pure aspartic acid. An irregular surface with a few pores was indicated by SEM. PXRD revealed the amorphous state of the developed hydrogels and confirmed the reduction in the crystallinity of the unreacted aspartic acid by the formulated hydrogels. An increase in gel fraction was observed with the increasing concentration of aspartic acid, acrylic acid, and ethylene glycol dimethacrylate due to the availability of a high amount of free radicals. The porosity study was influenced by the various compositions of developed hydrogels. Porosity was increased due to the enhancement in the concentrations of aspartic acid and acrylic acid, whereas it decreased with the increase in ethylene glycol dimethacrylate concentration. Similarly, the pH-responsive properties of hydrogels were evaluated by dynamic swelling and in vitro drug release studies at two different pH levels (1.2 and 7.4), and a greater dynamic swelling and acetaminophen release were exhibited at pH 7.4 as compared to pH 1.2. An increase in swelling, drug loading, and drug release was seen with the increased incorporation of aspartic acid and acrylic acid, whereas a decrease was detected with the increase in the concentration of ethylene glycol dimethacrylate. Conclusively, the formulated aspartic acid-based hydrogels could be employed as a suitable nonactive pharmaceutical ingredient for the controlled delivery of acetaminophen.

## 1. Introduction

The drug delivery system (DDS) is considered as a promising method to control postoperative inflammation [1], but still a lot of work is needed in order to enhance the effectiveness, biocompatibility, and controlled release of the drug for a prolonged time [2]. The use of biodegradable polymers has been increased specifically in DDSs because a biodegradable polymer-based drug delivery system does not require the removal of the polymers from the body, even after the treatment. They are degraded into physiologically occurring composites that are extracted readily from the body [3,4].

Hydrogels are three-dimensional structure networks, with a water holding capability of 10–20% in a random range up to a thousand times its dry weight [5,6,7]. A great interest has been shown recently in the development and use of stimuli-responsive smart hydrogels because they can show a response to physical, chemical, and environmental stimuli, i.e., temperature [8], pH [9], light, electric fields [10], and magnetic fields [11]. A key role is played by stimuli-responsive hydrogels in genes and controlled drug delivery systems, chemical/bioseparations [12], and sensors/actuators. Among the stimuli-responsive smart hydrogels, pH-responsive hydrogels are the most studied hydrogels that are used for the delivery of active compounds to the target areas of the gastrointestinal tract [13].

Aspartic acid (APA) is a biodegradable and synthetic polymer that contains amino groups or a large number of free carboxylic groups based on a natural amino acid [14]. Its solubility in water is high, whereas its toxicity is very low. Good biodegradability, high solubility, and low toxicity are the main characteristics that enabled this polymer to be used widely in detergents, cleaning products, and especially in controlled drug delivery systems [15]. APA contains a number of free carboxylic groups that protonate and deprotonate at low and high pH values. Due to protonation, the charged density is decreased because of the conjugate’s formation with counter ions through strong hydrogen bonding, and hence, low swelling is detected at a low pH. In the case of deprotonation, the charge density is increased, which produces strong electrostatic repulsive forces, and as a result, high swelling is exhibited at a high pH. This all leads to a polyelectrolyte effect, which stems from the ionization of carboxylic groups [16,17]. Negative charges are produced throughout the network due to ionization/deprotonation, which result in the endorsement of long chains and the globule-to-coil transition [18]. Acrylic acid (ACA) is a synthetic pH-sensitive monomer and its swelling index is mainly reliant on the pH of the environment. ACA contains carboxylic acid (functional group), which is associated strongly with the water molecules, and hence, the equilibrium swelling is greatly influenced by the ionic strength and pH of the swelled solution [19]. Furthermore, ACA forms corresponding esters when reacted in the presence of an alcohol. Similarly, ACA also plays an important role in the manufacturing of diapers, plastics, adhesives, paints, nail varnishes, floor polishes, coatings, etc. [20].

Acetaminophen (ACMP) is an odorless, slightly bitter, white crystalline powder. Its solubility in ether and water is low, but high in organic solvents such as ethanol and methanol. ACMP decreases the prostaglandin formation that is concerned with fever and pain processes by inhibiting the cyclooxygenase enzyme (COX-3). ACMP is used in relieving fever, pain, headache, and other moderate aches as it has an analgesic and antipyretic activity [21]. In the United States and Europe, people mostly take ACMP for relieving pain and fever. The World Health Organization approved ACMP as one of the most effective, essential, and safe medicines used in the management of pain and fever. Tylenol and Panadol are the trade names of the paracetamol (acetaminophen) available on the market [22]. The half-life of ACMP is 5.4 h [23]. ACMP is available in capsule, tablet, liquid, and suppository formulations [24]. The recommended dose of ACMP is 325–650 mg four times a day, whereas a single dose is 1 g. In the United States and Europe, the recommended dose of ACMP varies from 10 to 15 mg/kg/doses every 4 to 6 h. Taking such high doses of ACMP once, twice, or multiple times in a day generates GI problems, especially hepatotoxicity, and skin rashes in some patients [24,25,26]. It also reduces the patient compliance. Hence, different drug carrier systems are employed with the purpose of carrying the therapeutic agents to the specific areas in the body and overwhelming the harmful adverse effects. Desai and his coworkers prepared chitosan- and tripolyphosphate-based microspheres by the spray-drying method and reported a sustained release of ACMP for 6 h at pH 7.4 [27]. Similarly, Samanta et al. (2014) developed a semi-interpenetrating network of hydrogels of sodium alginate and polypolyacrylamide and reported a sustained release of ACMP for 6 h [28]. However, a new polymeric drug carrier system is still needed to overcome the limitations of the ACMP that are generated due to its frequent multiple-dose administration. Due to their unique properties, hydrogels are considered to be the most appropriate carrier for the controlled drug delivery systems [29,30].

The literature reveals that both APA and ACA play an important role in the development of various carrier systems, which could be used for the sustained/controlled delivery of drugs. Liu and his coworkers prepared a pH-sensitive interpenetrating polymer network of hydrogels of poly(aspartic acid) and polyvinyl alcohol and demonstrated the controlled release of naproxen sodium for 15 h [31]. Similarly, Liu et al. (2011) developed a semi-interpenetrating polymer network of hydrogels based on starch and poly(aspartic acid) for the targeted delivery of 5-fluorouracil to the colon up to 13 h [32]. The novelty of the current study is based on the crosslinking of APA with ACA by ethylene glycol dimethacrylate in the presence of an initiator. Researchers have focused recently on the use of APA, especially in controlled drug delivery systems, due to its unique properties including high aqueous solubility, excellent biodegradability, and negligible toxicity. Due to its pH-responsive nature, the introduction of APA with other reagents has been increased, particularly in the preparation of macroparticulate drug delivery systems such as hydrogels. The pH sensitivity of APA is increased with the enhancement in the pH of the medium due to the existence of COOH groups, which leads to deprotonation at high pH values. Similarly, ACA is a pH-sensitive and hydrophilic monomer, and is employed broadly in the synthesis of various pharmaceutical products. An increase is observed in the pH sensitivity, swelling degree, and drug loading and drug release rate of the fabricated carrier system by the incorporation of ACA with APA. Therefore, the recent crosslinking of APA and ACA has facilitated the developed hydrogels to swell highly at upper pH values. Thus, the developed pH-sensitive hydrogels have the maximum swelling and drug release at high pH values. The main advantage of this system is that it is not only limited to the controlled release of the drug for up to 24 h, but it also protects the stomach from the drug’s adverse effects and also protects the drug itself from the stomach acidity. Thus, comparing the previous published research work with the recent fabricated hydrogels, we can demonstrate that developed hydrogels can be used as an ideal drug delivery system for the controlled delivery of ACMP, particularly for those experiencing gastric acidity problems.

Hence, we developed the poly(aspartic acid-graft-poly(acrylic acid) hydrogels for oral controlled delivery of ACMP. The hydrogels constructed as a result of the crosslinking of APA and ACA have the potential to prolong the release of ACMP due to their pH-sensitive nature, which enabled the hydrogels to swell highly at a high pH as compared to a low pH. Therefore, greater drug release was observed at a high pH value of 7.4. Hence, the release of ACMP was prolonged significantly for 24 h in a controlled way by the fabricated hydrogels.

## 2. Results and Discussion

### 2.1. Synthesis of Polymeric Hydrogels

**Figure 1:**
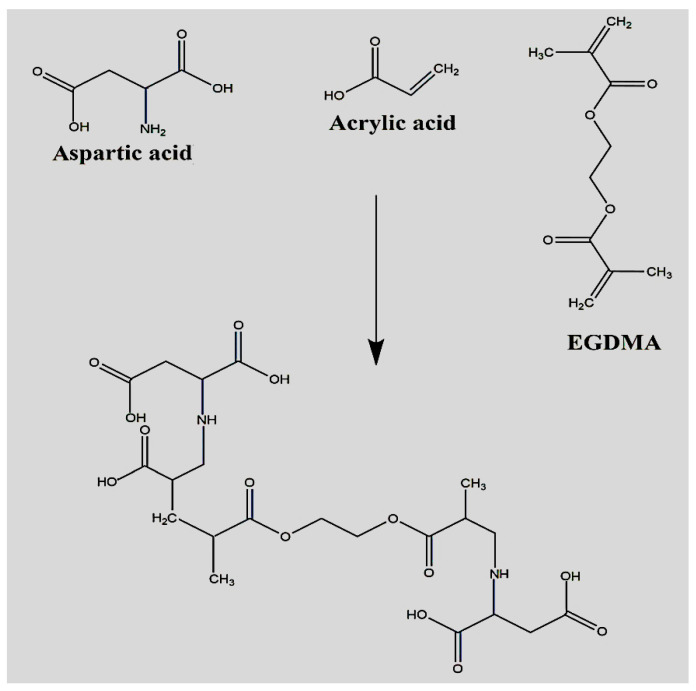
Proposed chemical structure of APA-g-PACA hydrogel.

**Figure 2:**
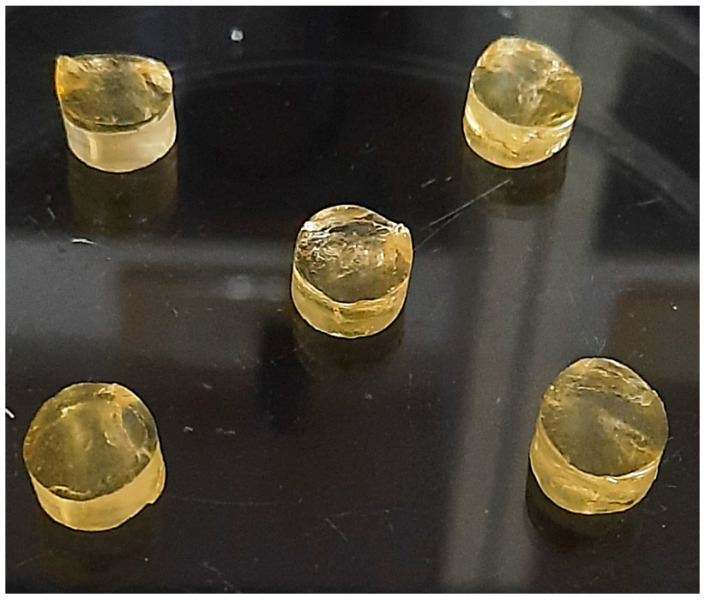
Physical appearance of APA-g-PACA hydrogel.

Different formulations of APA-g-PACA hydrogels were developed by the free radical polymerization method. The various concentrations of APA, ACA, and EGDMA are crosslinked in the presence of APS. An increase in stability and crosslinking density was observed with the incorporation of high concentrations of hydrogel contents. The proposed chemical structure and physical appearance of the dried APA-g-PACA hydrogels are indicated in Figure 1 and Figure 2, respectively. A series of studies was conducted for the prepared formulations to assess the different parameters of the fabricated hydrogels.

### 2.2. Sol-Gel Analysis

**Figure 3:**
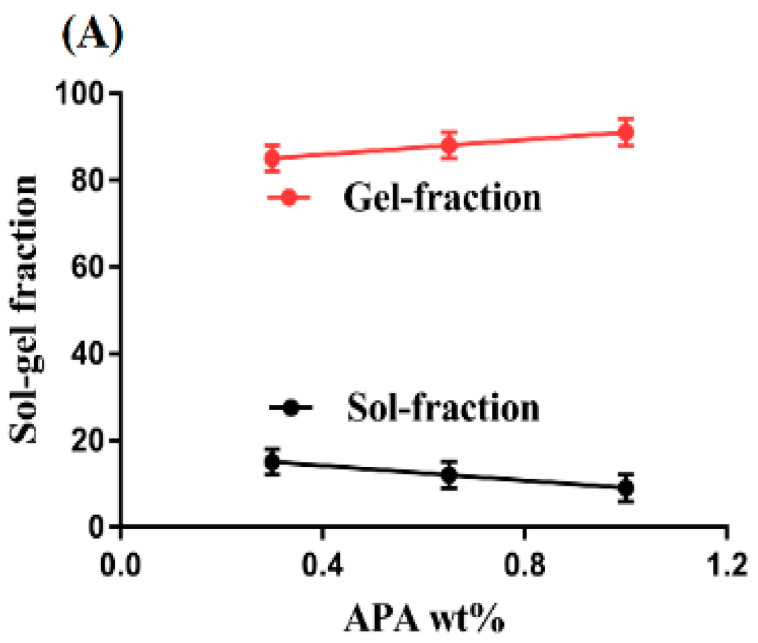

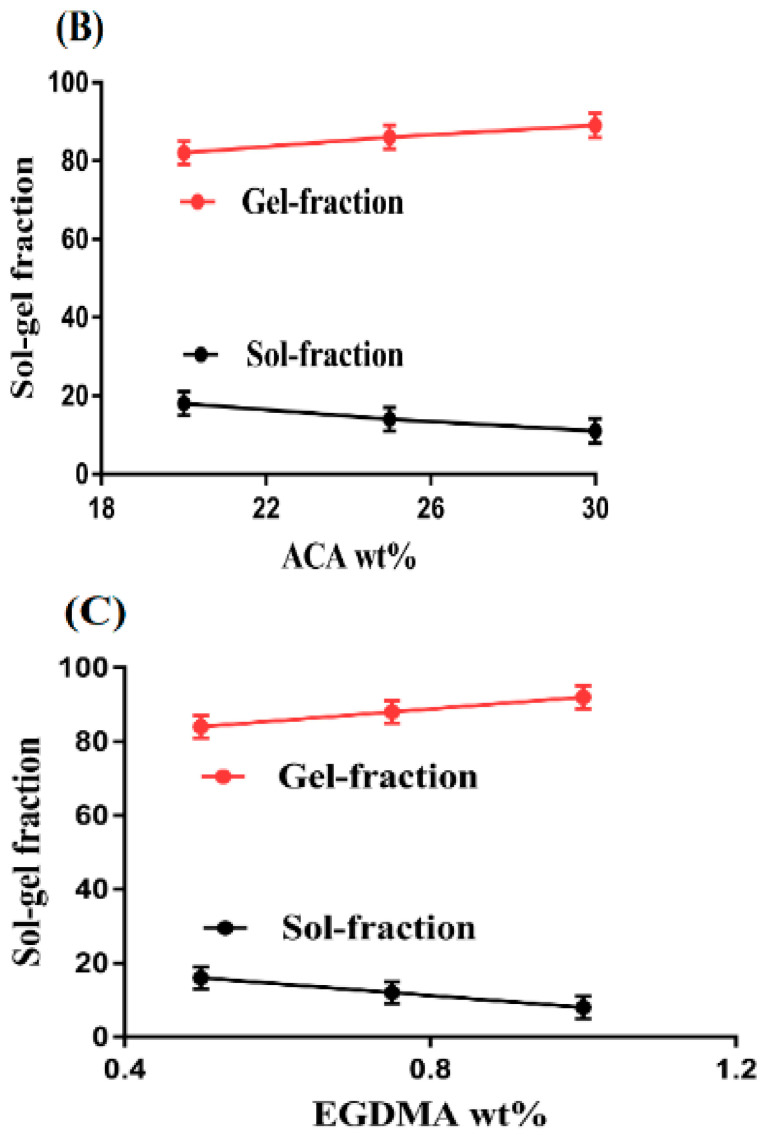
Effect of (**A**) APA, (**B**) ACA, and (**C**) EGDMA on the sol-gel fractions of APA-g-PACA hydrogels.

Polymerization of APA, ACA, and EGDMA resulted in the development of APA-g-PACA hydrogels. Hydrogels contain two fractions, (a) the sol fraction and (b) the gel fraction. Sol is the soluble uncrosslinked fraction of the hydrogels, whereas gel is the insoluble crosslinked fraction. When the hydrogel contents, i.e., APA, ACA, and EGDMA, are polymerized during the polymerization reaction, then some parts of the contents are not crosslinked properly due to the excess quantity of one of the reagents, which leads to the formation of the sol fraction. A drop in the sol fraction is observed with the increasing concentration of APA, ACA, and EGDMA, as shown in Figure 3A–C [33]. The gel fraction is the result of the crosslinking of hydrogel contents. It is an insoluble and crosslinked fraction of hydrogels. An increase in the gel fraction is seen with the increasing concentration of APA (Figure 3A) due to the high and rapid polymerization reaction between the APA and ACA. When the concentration of APA is high, a high number of free radicals are available for ACA, and thus, a rapid polymerization process is initiated. This all leads to escalation in the gel fraction. Similarly, the gel fraction is increased with the increasing concentration of ACA (Figure 3B). The higher the concentration of ACA, the greater the polymerization process is due to the fast crosslinking between APA and ACA, and thus, the greater the gel fraction is. Like other hydrogel contents, the gel fraction is increased with the increase in EGDMA concentration (Figure 3C). Crosslinking between the APA and ACA occurs very rapidly with the increase in the concentration of EGDMA. Therefore, a high concentration of EGDMA leads to fast crosslinking, and thus, a high, dense network of hydrogels is formed [34,35]. Khanum et al. (2018) prepared HPMC-g-poly(AMPS) hydrogels and demonstrated a high gel fraction with the increasing concentration of hydrogel contents [36]. Hence, we could conclude that the enhancement of the gel fraction occurs with the increase in APA, ACA, and EGDMA concentrations.

### 2.3. Fourier Transform Infrared (FTIR) Analysis

**Figure 4:**
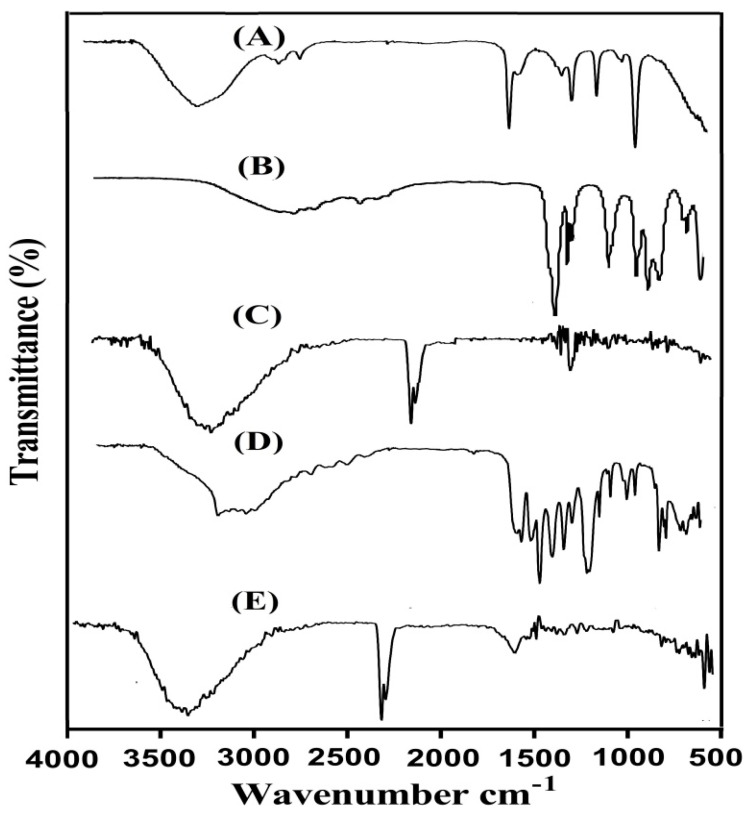
FTIR spectra of (**A**) APA, (**B**) ACA, (**C**) unloaded APA-g-PACA hydrogels, (**D**) ACMP, and (**E**) drug-loaded APA-g-PACA hydrogels.

FTIR spectra were obtained for APA, ACA, the unloaded APA-g-PACA hydrogels, ACMP, and the drug-loaded APA-g-PACA hydrogels, as indicated in Figure 4A–E. Two peaks are assigned by the FTIR spectrum of APA (Figure 4A) at 1413 and 3308 cm^−1^, representing the symmetric stretching vibration of carboxylate and OH groups, whereas peaks at 3452, 1557, and 1514 cm^−1^ represent the stretching vibration of N-H. Similarly, the peak at 1714 cm^−1^ indicates the absorption peak of C=O of the -COOH functional group. The same FTIR spectra of APA was also demonstrated by Zhao et al. (2006), which further supports our hypothesis [14]. Likewise, two prominent peaks are revealed by FTIR spectra of ACA (Figure 4B) at 2992 and 1692 cm^−1^, indicating the stretching vibration of OH and –C=O bending of the carboxylic group, whereas the stretching vibration of –C–C is indicated by a broad band at 1296 cm^−1^ [37]. A change is seen in the position of different bands of APA and ACA in FTIR spectra of unloaded APA-g-PACA hydrogels (Figure 4C). Characteristic bands of APA at 1413 and 1557 cm^−1^ are changed to 1452 and 1584 cm^−1^ bands in unloaded APA-g-PACA hydrogels. Similarly, peaks of ACA are also modified from 1296, 1692, and 2992 cm^−1^ to 1328, 1698, and 3020 cm^−1^, respectively. Some bands of APA and ACA are misplaced, whereas a few new bands are developed. The modification, misplacing, and formation of new peaks indicate the formulation of APA-g-PACA hydrogels and reveal the overlapping of ACA on the backbone of APA. The FTIR spectra of ACMP (Figure 4D) indicate the aromatic stretching vibration of C–C by peaks at 1450, 1530, and 1636 cm^−1^, whereas peaks at 1525, 1571, and 1582 cm^−1^ represent the stretching vibration of C–N [38]. Characteristic peaks of ACMP are revealed at 3460 and 3170 cm^−1^, representing the stretching vibration of the N-H (amide) group and free OH group. A slight modification is observed in the position of drug bands in the FTIR spectra of drug-loaded APA-g-PACA hydrogels (Figure 4E). The characteristic bands of ACMP at 1450, 1582, and 1636 cm^−1^ are modified slightly to 1463, 1610, and 1660 cm^−1^ in loaded APA-g-PACA hydrogels. Thus, we can conclude from the discussion that no interaction occurred between the drug and hydrogel networks [39].

### 2.4. Dynamic Swelling Studies

#### 2.4.1. Effect of pH on Swelling

**Figure 5:**
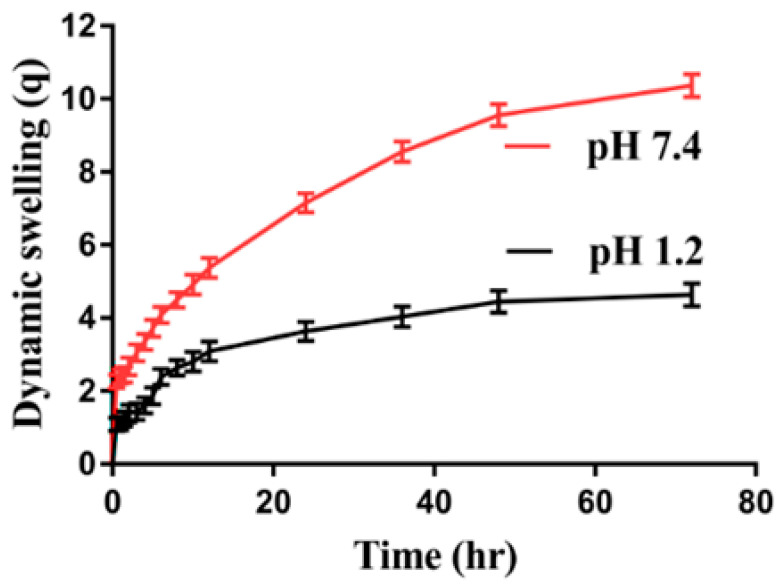
Effect of pH on dynamic swelling of APA-g-PACA hydrogels.

In order to understand the pH-responsive nature of the developed hydrogels, dynamic swelling at an acidic and basic medium (i.e., pH 1.2 and 7.4) was performed as indicated in Figure 5. pH greatly affects the dynamic swelling of hydrogels at both an acidic and basic medium. Greater swelling is observed at pH 7.4 as compared to pH 1.2 due to the deprotonation of functional groups of APA and ACA. APA contains COOH and NH groups, whereas ACA consists of COOH groups, which protonate at a lower pH of 1.2. Strong hydrogen bonding is formed among the functional groups of the APA and ACA and form conjugates with counter ions, which results in shrinkages of the hydrogel network, and thus, mostly low swelling is seen at pH 1.2. Contrary to pH 1.2, greater dynamic swelling is observed at pH 7.4 due to the deprotonation of the functional groups of the APA and ACA. The charge density among the COOH and NH groups of the APA is increased, which generates high, strong electrostatic repulsive forces, and as a result, swelling is increased. Similarly, the charge density of COOH groups of the ACA is increased because the pKa value of COOH groups of the ACA is near 4. Hence, with the increase in the pH of the medium from the lower to upper value, the charge density of the COOH groups increases, and as a result, the swelling increases due to the formation of strong electrostatic repulsive forces. Another reason is the decrease in hydrogen bonding. Hence, maximum swelling is seen at pH 7.4 as compared to pH 1.2 [40,41].

#### 2.4.2. Effect of APA/ACA/and EGDMA on Swelling

Dynamic swelling is also affected by the various concentrations of APA, ACA, and EGDMA at both the acidic and basic medium, as shown in Table 1. An increase in swelling is revealed with the increase in concentration of APA (Table 1). COOH and NH groups of the APA are highly generated with the increase in APA concentration, due to which a high charge density is generated, and as a result, an increase in dynamic swelling is perceived [41]. Similarly, an increase in the generation of the COOH group is observed with the increase in the ACA concentration, which also leads to a high charge density and strong repulsive forces, and as a result, an increase in swelling is observed (Table 1) [42,43]. Contrary to the polymer and monomer, a decrease in the swelling is perceived with the increasing concentration of EGDMA (Table 1). A possible reason for this is the hard bulk density of the hydrogels, which enhances as the concentration of EGDMA is increased. The hard, tight networks of the hydrogel decrease the pore size of the hydrogel, which causes a decrease in water penetration into the hydrogel networks, and as a result, a decrease in dynamic swelling is observed [44,45,46,47].

### 2.5. Polymer Volume Fraction

Polymer volume fraction analysis was conducted at both the acidic and basic medium (i.e., pH 1.2 and 7.4) for all APA-g-PACA hydrogel formulations as indicated in Table 1. The polymer volume fraction is seen to be low at the basic medium of pH 7.4 as compared to the acidic medium of pH 1.2. The hydrogel contents influence the polymer volume fraction highly, as a reduction is observed in the polymer volume as the concentration of APA and ACA is enhanced at both pH values. Unlike other hydrogel contents, the polymer fraction is increased as the concentration of EGDMA is increased. The reason can be associated with the swelling degree of the formulated hydrogels. The low polymer volume fraction at pH 7.4 and high at 1.2 reveals the substantial swelling and obvious expansion capability of the formulated hydrogels.

### 2.6. Thermogravimetric Analysis (TGA)

**Figure 6:**
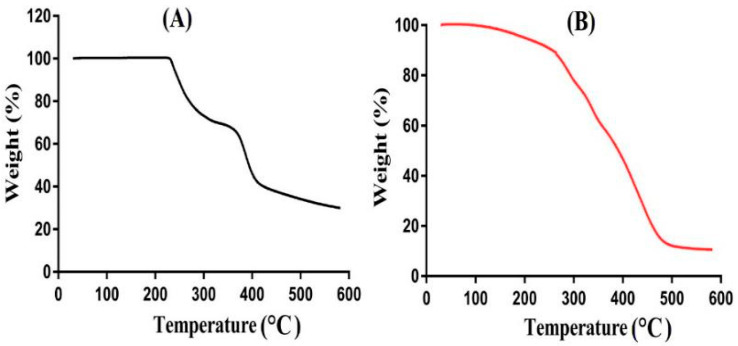
TGA of (**A**) APA and (**B**) APA-g-PACA hydrogels.

TGA was conducted for the purpose of evaluating and understanding the thermal stability of the hydrogel formulation and its content, i.e., APA, as shown in Figure 6A,B. TGA of APA (Figure 6A) reveals a weight reduction of 30% until the temperature reaches 312 °C. Further weight reduction of approximately 15% is observed as the temperature approaches 390 °C. A rapid reduction in weight is seen as the temperature was enhanced further, and then, APA degradation started at 408 °C due to the degradation of carboxyl and amino groups [48]. The TGA of APA-g-PACA hydrogels (Figure 6B) indicates that the degradation half-life of developed hydrogels (t1/2 = 488 °C) is greater than the degradation half-life of APA, i.e., APA (t1/2 = 408 °C). This means that the thermal stability of formulated hydrogels is greater than that of APA. A weight reduction of 12% is observed as the temperature reaches 270 °C, and a further weight reduction of 30% is followed when the temperature approaches 330 °C due to the degradation of carboxylic and amino groups of APA. Finally, at 488 °C, the degradation of formulated hydrogels starts and keeps going until the entirety of the formulated hydrogels is degraded. We can conclude from this discussion that formulated hydrogels are more thermally stable than the pure, unreacted polymer APA. B. Singh et al. (2019) prepared drug-loaded Carbopol-based hydrogels and demonstrated high thermal stability for the developed hydrogels as compared to their unreacted contents [49].

### 2.7. Differential Scanning Calorimetry (DSC) Analysis

**Figure 7:**
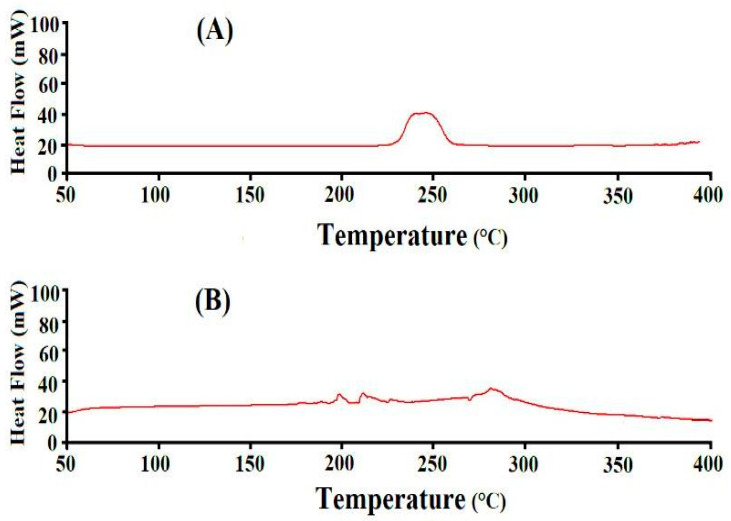
DSC of (**A**) APA and (**B**) APA-g-PACA hydrogels.

DSC analysis was conducted to understand the thermal stability of APA and formulated hydrogels, as indicated in Figure 7A,B. An endothermic peak is seen by the DSC of APA (Figure 7A) at 251 °C, whereas an exothermic peak is assigned at 260 °C [50]. Likewise, two exothermic peaks are seen at 190 °C and 275 °C by the DSC of APA-g-PACA hydrogels (Figure 7B). A modification is detected in the endothermic and exothermic peaks of APA that changed from 251 and 260 °C to 263 and 275 °C in APA-g-PACA hydrogels, showing the high thermal stability of the formulated hydrogels as compared to unreacted APA. This all indicates that APA, ACA, and EGDMA are successfully polymerized and formulated a stable network of hydrogels for the controlled delivery of ACMP. Khan et al. (2020) also demonstrated the same results as our study [51].

### 2.8. Percent Porosity

**Figure 8:**
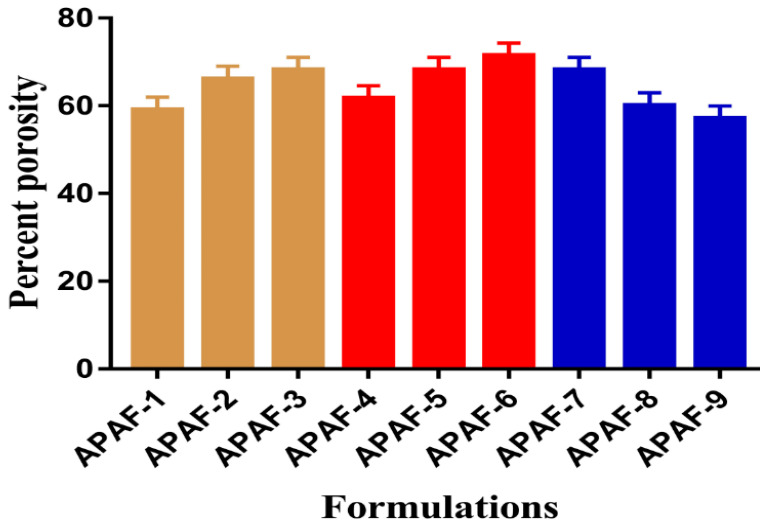
Percent porosity of APA-g-PACA hydrogels.

Porosity plays an important role in the swelling, drug loading, and percent of drug release from the developed hydrogels. Swelling is greater if the surface of the hydrogels is porous because a greater quantity of water penetrates through the pores into the polymeric network, and as a result, high swelling is perceived, which leads to the maximum drug loading and release. An increase in the percent porosity is seen with the increase in the concentration of APA and ACA of the developed hydrogels, as indicated in Figure 8. Due to high viscosity, the bubbles of the reaction mixture are restricted from evaporating, and thus, interconnected channels are generated that lead to high porosity. As opposed to the polymer and monomer, porosity is decreased with the increasing concentration of EGDMA (Figure 8) due to the strong, crosslinked bulk density and hard structure, which influence the flexibility of the drug [52].

### 2.9. Morphology of Hydrogels

**Figure 9:**
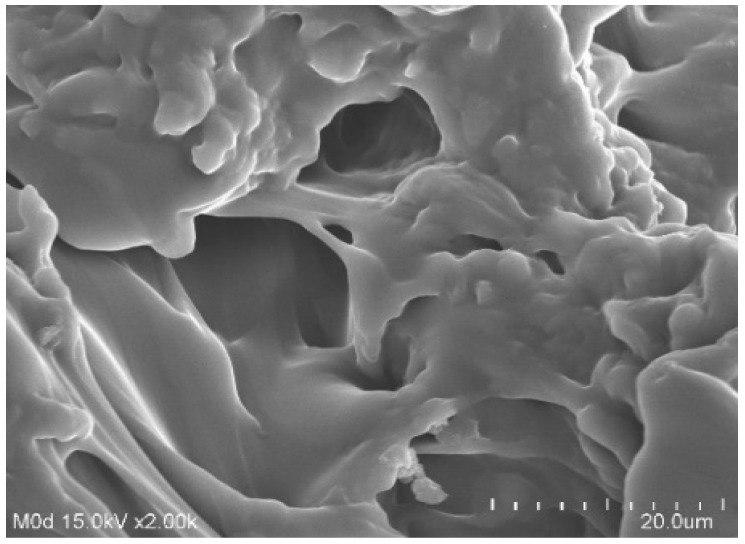
Scanning electron microscopy of APA-g-PACA hydrogels.

The surface morphology of the formulated hydrogels was analyzed by scanning electron microscopy as indicated in Figure 9. An uneven surface with a few pores is shown by the formulated hydrogels, indicating that there is water penetration into the formulated hydrogels through the pores, due to which potential swelling is perceived by the developed hydrogels. Hence, the greater the porosity of the formulated hydrogels, the higher the swelling, loading, and release of drug [28] and vice versa.

### 2.10. Drug Loading

The loading of the drug by the formulated hydrogels is dependent on the swelling of hydrogels. The greater the swelling, is the greater the drug loading and vice versa. Like swelling, drug loading is also influenced by the different concentrations of hydrogel contents. An increase in the drug loading is seen with the increasing concentration of the APA (Table 1). The appropriate reason is the generation of high repulsive forces by the functional groups of APA that lead to greater swelling, and thus, a greater quantity of drug is loaded. Similarly, high amounts of water penetrate into the hydrogel network due to greater swelling and because a high charge density of carboxylic groups of the ACA is generated, which results in the enhancement in the ACA concentration (Table 1). Contrary to APA and ACA, a decrease in the loading of the drug is detected with the enhancement in the EGDMA concentration (Table 1). The main reason is the high bulk density of the hydrogels that decreases the porosity of the network, and hence, a decrease in swelling and drug loading is observed ultimately [53].

### 2.11. Powder X-ray Diffraction (PXRD) Study

**Figure 10:**
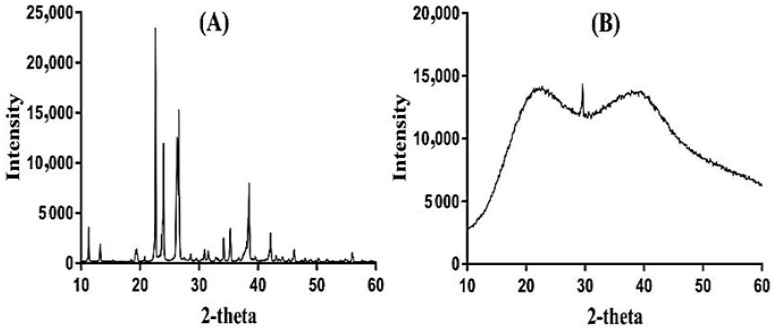
PXRD of (**A**) APA and (**B**) APA-g-PACA hydrogels.

PXRD was conducted for APA and APA-g-PACA hydrogels in order to analyze their crystallinity, as shown in Figure 10A,B. PXRD of APA reveals prominent peaks at 2θ = 21.10°, 24.42°, 28.53°, and 41.63° (Figure 10A) that indicate the crystallinity of APA. All the crystalline peaks of the APA disappear or are reduced in the PXRD of APA-g-PACA hydrogels (Figure 10B), which determines that the crystallinity of the pure polymer disappears or is reduced by the formulated hydrogels, and hence, an amorphous nature is exhibited by the formulated hydrogels. The results reveal the formation of a strong chemical bond between the APA and ACA during the polymerization reaction, due to which the crystallinity of the pure polymer disappears or is reduced. Lee and his coworkers prepared a copolymer-based hydrogel and stated a decline in the high, intense crystalline peaks of the excipients as shown by the PXRD of developed hydrogels [54], which further supports our hypothesis.

### 2.12. In Vitro Drug Release Study and Kinetics

#### 2.12.1. Effect of pH on Drug Release

**Figure 11:**
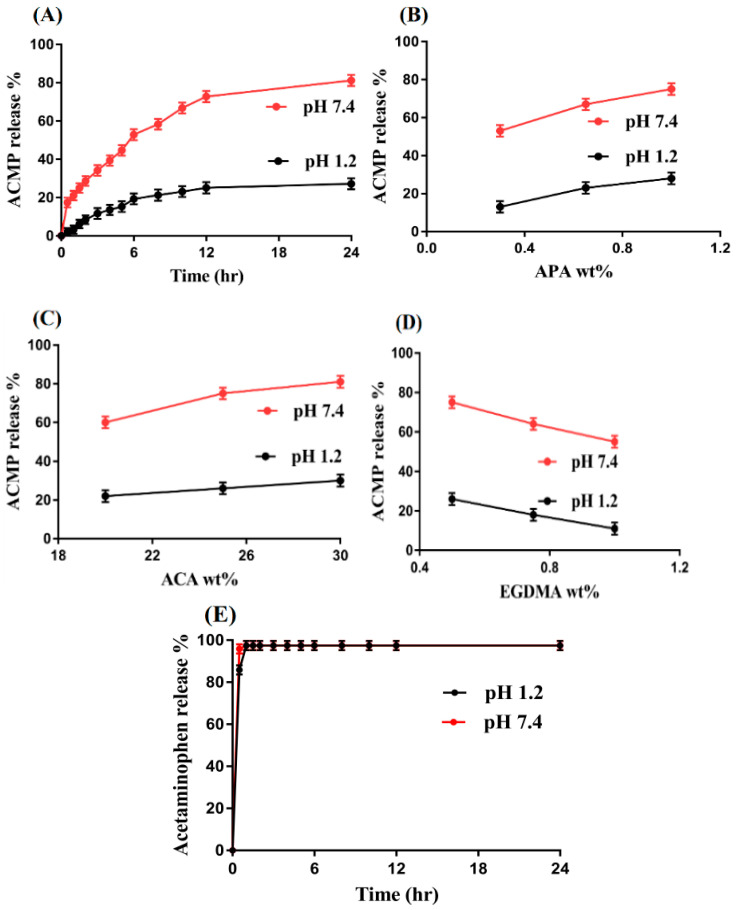
Effect of (**A**) pH, (**B**) APA, (**C**) ACA, (**D**) EGDMA, and (**E**) commercial product on percent of ACMP release from APA-g-PACA hydrogels.

As with swelling, in vitro drug release is influenced highly by the pH of both the acidic and basic medium. A high percent of drug release is observed at pH 7.4 as compared to pH 1.2, as shown in Figure 11A. The low release of the drug at pH 1.2 is due to the protonation of functional groups of the polymer APA and monomer ACA. The functional groups (COOH and NH) of the APA and (COOH) ACA are protonated and form a conjugate with counter ions by strong hydrogen bonding, due to which the charge density is decreased, and as a result, low swelling is exhibited, and thus a low percent of drug release is detected. On the other hand, as the pH is changed from 1.2 to 7.4, deprotonation of the COOH and NH groups of the APA and COOH groups of the ACA occurs, due to which the charge density is increased and electrostatic repulsive forces are produced, which result in high swelling, and thus, the maximum amount of the drug is released [55,56].

#### 2.12.2. Effect of APA/ACA/EGDMA and the Commercial Product (Acetaminophen) on Drug Release

The percent of drug release is influenced highly by the hydrogel contents at both low and upper pH values, as shown in Figure 11B–D. The charge density of the functional groups of both APA and ACA is increased as their concentration increases, and as a result, the percent of drug release is increased (Figure 11B,C) [57] and vice versa. A decrease is detected in the percent of drug release with the increasing concentration of the EGDMA (Figure 11D). The reason may be the high crosslinking and bulk density, which leads to a reduction in swelling, drug loading, and percent of drug release. The pore size is decreased due to the tight junction and hard network of hydrogels, and thus, a sufficient amount of water does not penetrate into the hydrogel network, causing the swelling, loading, and percent of drug release to decrease [58] and vice versa. Similarly, an in vitro drug release study was carried out for the commercial product acetaminophen (500 mg, YUNGSHIN PHARM IND. CO. LTD (lot number: M029)) at the same pH values (pH 1.2 and 7.4), as shown in Figure 11E. Drug releases from the commercial product happened very rapidly at both pH values. Within the initial 0.5 h, 85% of the drug is release at pH 1.2, whereas a drug release of 96% is detected at pH 7.4 within 0.5 h. Comparing the percent of drug release of the commercial product acetaminophen with fabricated hydrogels indicates that the drug release is sustained significantly by fabricated hydrogels over a long time in a controlled manner.

Various kinetic models such as zero-order, first-order, Higuchi, and Korsmeyer–Peppas models were computed to obtain release data in order to understand the drug release mechanism from the formulated hydrogels. “r” values determine the type of kinetic modeling exhibited by all formulations of the prepared system. “r” values of the first-order of kinetics are higher than the “r” values of all other respective models (Table 2), which confirmed that the prepared hydrogels follow the first-order of kinetic modeling. The type of diffusion is determined by “*n*” values, where a Fickian diffusion mechanism is *n* = 0.5 and non-Fickian or anomalous is *n* > 0.5. The “*n*” values are found within the range of 6506–0.9417, which determines the non-Fickian diffusion mechanism. APAF-1 is the exception that represents super case II transport because the “*n*” value is greater than 0.85 [59,60].

#### 2.12.3. Comparative Study of ACMP-Loaded APA-g-PACA Hydrogels with Other Delivery Systems for ACMP

The most commonly used method for the preparation of stimuli-sensitive hydrogels is the free radical polymerization method because this technique does not require any specific pressure and temperature. The polymerization process occurs very fast and leads to permanent crosslinking of the prepared gels, which are then used for multiple purposes [42].

A comparison of ACMP-loaded APA-g-PACA hydrogels with other used delivery systems for ACMP is made on the basis of the intended amount of loaded formulation for drug release, maximum % of drug release, and time for maximum % of drug release as indicated in Table 3. Keeping the given parameters, it is demonstrated in Table 3 that the properties of the developed hydrogels are mostly comparable with the previously reported delivery systems for ACMP. The main advantage of the developed hydrogels can be correlated with their pH-sensitive nature, which minimized the release of the drug at a low pH while maximizing at high pH values. Thus, those experiencing stomach acidity problems can be protected from stomach acidity by loading such drugs into this polymeric carrier system of hydrogels.

## 3. Conclusions

APA-g-PACA hydrogels were prepared successfully by the free radical polymerization of APA, ACA, and EGDMA. Sol-gel analysis determined the soluble uncrosslinked and insoluble crosslinked fractions of the formulated hydrogels. FTIR confirmed the grafting of ACA over the backbone of APA. TGA and DSC revealed that the thermal stability of the formulated hydrogels was greater than APA, which determines, basically, an increase in the thermal stability of the APA after crosslinking with other hydrogel contents. SEM indicated a rough and hard surface of the fabricated hydrogels. Maximum swelling and drug release at pH 7.4 compared to low swelling and drug release at pH 1.2 indicated the pH-responsive nature of the fabricated hydrogels. The maximum swelling and drug release at pH 7.4 were due to the deprotonation of functional groups of the polymer and monomer. An increase in porosity, drug loading, swelling, and drug release was observed with the increase in the concentrations of APA and ACA, whereas a decrease was seen with the incorporation of a high concentration of EGDMA. Similarly, a decrease in the crystallinity of APA by the formulated hydrogels was revealed by PXRD analysis. Hence, we can conclude from the reported results that the developed hydrogels could be used as a suitable carrier for the controlled drug delivery systems.

## 4. Materials and Methods

### 4.1. Materials

Acetaminophen was obtained from Sigma-Aldrich (St. Louis, MO, USA). Acrylic acid (ACA; purity = 98%, extra pure, MW = 72.06 g/mol) and aspartic acid (APA; purity = 99 plus%, MW = 133.10) were purchased from Acros (Carlsbad, CA, USA). Similarly, ethylene glycol dimethacrylate (EGDMA; purity = 98%, MW = 198.22 g/mol) was procured from Alfa Aesar (Tewksbury, MA, USA), whereas ammonium persulfate (APS; purity = 98%, MW = 228.21) was obtained from Showa, Tokyo, Japan.

### 4.2. Synthesis of Polymeric Hydrogels

A set of nine formulations with different concentrations of the polymer APA, monomer ACA, and crosslinker EGDMA were crosslinked in the presence of the initiator APS with its constant concentration for the preparation of aspartic acid-graft-poly(acrylic acid) (APA-g-PACA) hydrogels, created via the free radical polymerization technique as shown in Table 4. APA and APS are completely soluble in water; hence, the weighed amount of both APA and APS was dissolved in the required quantity of deionized distilled water separately. ACA and EGDMA were already in solution form. The solution of APS was added slowly into the APA solution and was stirred continuously. After a few minutes, the ACA solution was added dropwise into the stirred mixture of APA and APS. Finally, the EGDMA solution was poured into the mixture, which was stirred until a transparent solution was formed. The solution was then purged by nitrogen gas in order to remove dissolved oxygen from the transparent solution. The solution was poured into glass molds and placed in a water bath at 55 °C initially for 2 h. The temperature was then enhanced up to 65 °C for the next 22 h so that a compact structure of gel was formed. The formed gels were cut into 8 mm discs. A mixture of water and ethanol was used for washing the discs of hydrogel, which were placed at room temperature for 24 h. After that, the discs were placed in the vacuum oven at 40 °C for 7 days in order to dry the discs completely. The prepared discs of hydrogel were assessed for further studies.

### 4.3. Sol-Gel Analysis

The sol-gel study was accomplished for the fabricated hydrogels with the purpose of understanding the sol and gel fraction of the developed hydrogels. Therefore, the Soxhlet extraction technique was carried out where an accurately weighed hydrogel disc was placed in a round bottom flask containing a specific volume of deionized distilled water. The flask was attached to a condenser. The flow of water was kept constant at a temperature of 85 °C throughout the experiment. After 10 h, the extracted disc was removed and placed in a vacuum oven at 40 °C until completely desiccated [64]. Sol and gel fractions were estimated by the given formulas:

(1) $$\mathrm{Sol}\;\mathrm{fraction}\;\%=\frac{T_{1}-T_{2}}{T_{2}}\times 100$$

(2) Gel fraction=100−Sol fraction

where T_1_ specifies the initial weight of the dried disc of the hydrogels before the extraction process, and T_2_ shows the final weight of the dried disc of the hydrogels after the extraction process.

### 4.4. Fourier Transform Infrared (FTIR) Analysis

FTIR analysis was carried out for unreacted APA, ACA, the unloaded APA-g-PACA hydrogels, ACMP, and the drug-loaded APA-g-PACA hydrogels. The samples were ground up to the desire size and then placed in capped glass vials for FTIR analysis. After this, a Nicolet 380 FTIR (Thermo Fisher Scientific, Ishioka, Japan) was used for the sample analysis within the range of 4000–500 cm^−1^ [65].

### 4.5. Dynamic Swelling Studies

In order to investigate the pH sensitivity of all formulations of the fabricated hydrogels at two different swelling pH mediums (pH 1.2 and 7.4), dynamic swelling studies were conducted. The accurate weight of the dried hydrogel disc was taken and dipped in 100 mL of pH 1.2 and 7.4 phosphate-buffer solution at 37 °C. After a regular interval of time, the hydrogel disc was removed from the referred medium, blotted with filter paper to eliminate spare fluid, and weighed on the weighing balance again. This procedure was continued until a constant equilibrium weight of the hydrogel disc was obtained [66]. This study was conducted in triplicate. Dynamic swelling was calculated by the given equation:

(3) $$\left(q\right)=\frac{B_{2}}{B_{1}}$$

where q = dynamic swelling, B_1_ = initial weight of the hydrogel disc before swelling, and B_2_ = final weight of the hydrogel disc after swelling at time t.

### 4.6. Polymer Volume Fraction

The fraction of the polymer in the completely swelled state of the hydrogel is recognized as the polymer volume fraction. This experiment was performed for all formulations of the formulated hydrogels and denoted as V2,s. The polymer volume fraction was determined by using the equilibrium volume swelling (Veq) data at both pH 1.2 and 7.4. Hence, the given formula was used for the polymer volume fraction determination:

(4) $$V2,s=\frac{1}{Veq}$$

### 4.7. Thermogravimetric Analysis (TGA)

The TGA study (TA Instruments, PerkinElmer, Simultaneous Thermal Analyzer (STA) 8000) was conducted for APA and the APA-g-PACA hydrogels to understand and evaluate the thermal stability of the unreacted APA and fabricated hydrogels. Hence, an accurate amount of 0.5–4 mg of the sample was taken in an open pan attached to a microbalance. The samples were heated from 40 to 600 °C with a heating rate of 20 °C/min. The nitrogen flow was maintained constant at 20 mL/min throughout the experiment [67].

### 4.8. Differential Scanning Calorimetry (DSC) Analysis

The DSC study (PerkinElmer, DSC 4000) was performed for APA and the APA-g-PACA hydrogels in order to know the thermal stability of APA and formulated hydrogels; then, the thermal stability of the unreacted APA was compared with the developed hydrogels. Therefore, samples of 0.3–5 mg were taken in an aluminum pan and investigated with a temperature of 50–400 °C and a heating rate of 20 °C/min. Nitrogen flow was maintained at 20 mL/min throughout the study [68].

### 4.9. Percent Porosity

The percent porosity was analyzed by the solvent replacement method. Hence, weighed hydrogel discs (*M*_1_) were submerged in absolute ethanol (purity > 99.9%) for 72 h. After that, the discs of all hydrogel formulations were eliminated, cleaned with filter paper to take out the extra solvent, and weighed again (*M*_2_). Likewise, the diameter and thickness of the hydrogel discs were measured [69]. This formula was used for the calculation of percent porosity:

(5) $$\mathrm{Porosity}\;\mathrm{percentage}\;(\%)=\frac{M_{2}-M_{1}}{\rho V}\times 100$$

where *ρ* shows the density of absolute ethanol, whereas V indicates the volume of the swelled hydrogel.

### 4.10. Morphology of Hydrogels

Scanning electron microscopy (SEM) was used for the surface analysis of fabricated hydrogels. Hence, a precise quantity of weighed hydrogels was fixed on a piece of double-adhesive tape and trapped to an aluminum stub. Gold was coated on the stubs with the help of gold sputter under an organic atmosphere. After this, the samples were scanned, and the surface morphology was observed by photomicrographs that were recorded during the scanning of samples [70].

### 4.11. Drug Loading

An absorption and diffusion method was employed for the loading of the drug by the developed hydrogels. Dried hydrogel discs of accurate weight of all formulations were placed in 1% ACMP solution of a 100 mL phosphate buffer with a pH of 7.4 for 72 h. The selection of the proper solvent for drug loading is very important, because solubility of the drug and swelling of the formulated hydrogels should be higher in the solvent used for drug loading. Hence, the dipped hydrogel discs were removed from the drug solution after achieving equilibrium swelling. Loaded hydrogel discs were washed by distilled water. The loaded discs of hydrogel were placed at 40 °C in a vacuum oven.

Quantification of drug loading by the hydrogels was performed via two methods. The first was the extraction method, where the weighed loaded discs of hydrogel were placed in 25 mL of fresh buffer solution for a specific period of time, and samples were collected. This process was continued until all the drug was eliminated from the loaded discs of hydrogel. The drug contents in the collected samples were then analyzed by using a UV–vis spectrophotometer (U-5100, 3J2-0014, Tokyo, Japan) at a wavelength (λmax) of 243 nm. The other method was the weight method. In this method, the precise weight of unloaded hydrogel discs was subtracted from the accurate weight of drug-loaded hydrogel discs [71].

Quantity of Drug loaded = W_M_ − W_N_ (6)

where W_M_ = weight of dried loaded hydrogel disc, and W_N_ = weight of dried unloaded hydrogel discs.

### 4.12. Powder X-ray Diffraction (PXRD) Analysis

To identify the crystallinity of the APA and formulated hydrogels, PXRD (XRD-6000 SHIMADZU, Tokyo, Japan) was conducted. The diffraction angle was kept within the range of 10–60° at a rate of 2° 2θ/min [72].

### 4.13. In Vitro Dissolution and Kinetics

The pH-responsive release of ACMP from the formulated hydrogels at both lower and upper pH values was examined by an in vitro dissolution study. Similarly, the dissolution study was performed for the commercial product acetaminophen (500 mg, YUNGSHIN PHARM IND. CO. LTD (lot number: M029)) at the same pH values. A buffer medium of 900 mL of both pH 1.2 and 7.4 was used for the drug release study. Therefore, accurate drug-loaded hydrogel discs were immersed in the respective buffer mediums by using the USP dissolution apparatus-II (Sr8-plus Dissolution Test Station) at a temperature of 37 ± 0.5 °C and 50 rpm. A sample of 5 mL was collected at a regular interval of time and a fresh medium of 5 mL was added back to keep the sink condition constant. The samples were then examined on the UV–vis spectrophotometer (U-5100, 3J2-0014, Tokyo, Japan) at a wavelength (λmax) of 243 nm. This experiment was conducted in triplicate.

Various kinetic models, i.e., zero-order, first-order, Higuchi, and Korsmeyer–Peppas models were computed for assessing the release data of different formulations of the formulated hydrogels to understand the drug release mechanism from the developed hydrogels [73].

## Figures and Tables

**Table 1:** Dynamic swelling, drug loading, and polymer volume fraction of APA-g-PACA hydrogels.

F. Code	Dynamic Swelling up to 72 h		Drug Loading (mg)/400 mg of Dry Gels		Polymer Volume Fraction	
	pH 1.2	pH 7.4	Weight method	Extraction method	pH 1.2	pH 7.4
APAF-1	3.63 ± 0.21	09.89 ± 0.18	93.3 ± 1.5	91.8 ± 0.9	0.275	0.101
APAF-2	3.92 ± 0.19	10.12 ± 0.12	98.2 ± 1.2	95.7 ± 1.6	0.255	0.098
APAF-3	4.53 ± 0.18	10.29 ± 0.24	105.4 ± 1.2	103.1 ± 1.8	0.220	0.097
APAF-4	3.77 ± 0.24	9.56 ± 0.27	81.2 ± 1.1	78.3 ± 1.1	0.265	0.104
APAF-5	4.53 ± 0.18	10.29 ± 0.24	105.4 ± 1.2	103.1 ± 1.8	0.220	0.097
APAF-6	4.70 ± 0.20	10.80 ± 0.26	112.8 ± 0.8	110.2 ± 1.1	0.212	0.092
APAF-7	4.53 ± 0.18	10.29 ± 0.24	105.4 ± 1.2	103.1 ± 1.8	0.220	0.097
APAF-8	3.67 ± 0.26	6.76 ± 0.21	96.3 ± 1.0	94.5 ± 1.3	0.272	0.147
APAF-9	3.41 ± 0.14	6.10 ± 0.13	86.5 ± 0.1	84.9 ± 0.9	0.293	0.163

**Table 2:** Kinetic modeling release of ACMP from APA-g-PACA hydrogels.

F. Code	Zero-Order	First-Order	Higuchi	Korsmeyer–Peppas	
	r^2^	r^2^	r^2^	r^2^	*n*
APAF-1	0.8587	0.9071	0.7013	0.8880	0.9417
APAF-2	0.9493	0.9631	0.9545	0.9697	0.8448
APAF-3	0.9635	0.9856	0.9760	0.9716	0.8260
APAF-4	0.9469	0.9491	0.9063	0.9094	0.6506
APAF-5	0.9635	0.9856	0.9760	0.9716	0.8260
APAF-6	0.9372	0.9757	0.9823	0.9726	0.7507
APAF-7	0.9635	0.9856	0.9760	0.9716	0.8260
APAF-8	0.9761	0.9893	0.9812	0.9858	0.7076
APAF-9	0.9912	0.9954	0.9422	0.9700	0.6864

**Table 3:** Comparison of drug-loaded APA-g-PACA hydrogels with other ACMP delivery systems.

S. No.	Formulation	Intended Quantity of Loaded Formulation for Drug Release (mg)	Maximum % of Drug Release	Time for Maximum % of Drug Release	Reference
1	Eudragit S100-based nanoparticles	50	28.31	12 h	[22]
2	Tramadol HCl and acetaminophen microparticles	531	99.5	12 h	[21]
3	Acetaminophen-loaded poly(L-lactide) microcapsules	80	83.50	24 h	[61]
4	Hydroxypropylmethylcellulose matrix tablets containing acetaminophen	-	100	8 h	[62]
5	Acetaminophen- and tramadol hydrochloride-loaded soft gelatin capsule	325	100	0.5 h	[63]
6	APA-g-PACA hydrogels	400	84.62	24 h	Current study

**Table 4:** Compositions of formulations of APA-g-PACA hydrogels.

Formulation Code	Polymer (APA) g/100 g	Monomer (ACA) g/100 g	Initiator (APS) g/100 g	Crosslinker (EGDMA) g/100 g
APAF-1	0.30	25	0.5	0.50
APAF-2	0.65	25	0.5	0.50
APAF-3	1.00	25	0.5	0.50
APAF-4	1.00	20	0.5	0.50
APAF-5	1.00	25	0.5	0.50
APAF-6	1.00	30	0.5	0.50
APAF-7	1.00	25	0.5	0.50
APAF-8	1.00	25	0.5	0.75
APAF-9	1.00	25	0.5	1.00
